# Intrahepatic Administration of Human Liver Stem Cells in Infants with Inherited Neonatal-Onset Hyperammonemia: A Phase I Study

**DOI:** 10.1007/s12015-019-09925-z

**Published:** 2019-12-02

**Authors:** Marco Spada, Francesco Porta, Dorico Righi, Carlo Gazzera, Francesco Tandoi, Ivana Ferrero, Franca Fagioli, Maria Beatriz Herrera Sanchez, Pier Luigi Calvo, Elisa Biamino, Stefania Bruno, Monica Gunetti, Cristina Contursi, Carola Lauritano, Alessandra Conio, Antonio Amoroso, Mauro Salizzoni, Lorenzo Silengo, Giovanni Camussi, Renato Romagnoli

**Affiliations:** 1 Department of Pediatrics, AOU Città della Salute e della Scienza di Torino, University of Torino, Piazza Polonia 94, 10126 Torino, Italy; 2 Department of Radiology, AOU Città della Salute e della Scienza di Torino, Torino, Italy; 3 General Surgery 2U, Liver Transplant Center, AOU Città della Salute e della Scienza di Torino, University of Torino, Torino, Italy; 4 Pediatric Oncoematology, AOU Città della Salute e della Scienza di Torino, University of Torino, Torino, Italy; 5 2i3T – Incubatore di Imprese dell’Università degli Studi di Torino, Torino, Italy; 6 Molecular Biotechnology Center, University of Torino, Torino, Italy; 7 Department of Medical Sciences, AOU Città della Salute e della Scienza, University of Torino, Corso Dogliotti 14, Torino, Italy; 8 Pediatric Anesthesia and Intensive Care, AOU Città della Salute e della Scienza, Torino, Italy

**Keywords:** Liver transplantation, Inborn errors of metabolism, Stem cells, Hepatocytes, Ammonia

## Abstract

Previous studies have shown that human liver stem-like cells (HLSCs) may undergo differentiation in vitro into urea producing hepatocytes and in vivo may sustain liver function in models of experimentally induced acute liver injury. The aim of this study was to assess the safety of HLSCs intrahepatic administration in inherited neonatal-onset hyperammonemia. The study was approved by the Agenzia Italiana del Farmaco on favorable opinion of the Italian Institute of Health as an open-label, prospective, uncontrolled, monocentric Phase I study (HLSC 01–11, EudraCT-No. 2012–002120-33). Three patients affected by argininosuccinic aciduria (patient 1) and methylmalonic acidemia (patients 2 and 3) and included in the liver transplantation list were enrolled. In all patients, HLSCs were administered by percutaneous intrahepatic injections (once a week for two consecutive weeks) within the first months of life. The first patient received 125,000 HLSCs x gram of liver/dose while the other two patients received twice this dose. No immunosuppression was administered since HLSCs *possess* immunomodulatory activities. None of the patients experienced infections, hyperammonemia decompensation, or other adverse events during the whole observation period. No donor specific antibodies (DSA) against HLSCs were detected. Patients were metabolic stable despite an increase (~30%) in protein intake. Two patients underwent liver transplantation after 19 and 11 months respectively, and after explantation, the native livers showed no histological alterations. In conclusion, percutaneous intrahepatic administration of HLSCs was safe in newborn with inherited neonatal-onset hyperammonemia. These data pave the way for Phase II studies in selected inherited and acquired liver disorders.

## Introduction

Long-term medical management of neonatal-onset hyperammonemia due to urea cycle disorders (UCDs) or organic acidemias (OAs) requires dietary restrictions, orphan medications and intensive clinical monitoring, which have a strong negative effect on the quality of life of patients. In spite of conventional treatment, acute metabolic decompensations, comorbidity, and disappointing deadly prognosis hamper the clinical course of these disorders [1]. To face this severe prognosis, liver transplantation has been growingly indicated in UCDs and OAs (mostly methylmalonic acidemia), with successful prevention of metabolic decompensations, improved clinical prognosis, and amelioration of quality of life [2–6]. In newborns, however, bridging strategies to liver transplantation are necessary to prevent metabolic sequelae in the pre-transplantation window and to optimize the overall clinical outcome. Hepatocyte transplantation was introduced in 1997 and attempted in various inborn errors of metabolism with transient metabolic effectiveness [7–9]. Either fresh or cryopreserved hepatocytes isolated from a liver donor were administered via the portal vein to correct the inherited enzyme deficiency. However, several issues complicated the feasibility and safety of liver transplantation in the clinical practice, including shortage of donor livers, the quality of cryopreserved cells, the need for invasive procedures to perform portal infusions, limited space in the native liver, the risk of extra-hepatic spread of transplanted hepatocytes (mostly to the lung), limited engraftment of transplanted cells and the need of immunosuppression to prevent rejection. These limitations led to an increased interest in the potential use of stem cells with hepatic differentiation capability [10].

Human liver stem-like cells (HLSCs) are a mesenchymal-like progenitor cell population with self-renewing capability derived from human adult liver tissue [11]. HLSCs express some hepatocyte markers such as albumin, cytokeratin 8 and alpha-fetoprotein, some mesenchymal stem cells markers (CD73, CD29, CD90, CD105) and some embryonic markers (Nanog, Oct 3/4, Musashi1) [11, 12]. In vitro*,* HLSCs showed multiple differentiation potentials, including differentiation into mature hepatocyte [12] and pancreatic islet-like organoid differentiation [13]. In vivo, HLSCs were shown to increase survival in a lethal model of fulminant liver failure and to restore liver function [12].

The main objective of this Phase I study in newborns suffering from inherited neonatal-onset hyperammonemia was that to assess the clinical safety of HLSCs intrahepatic administration The secondary objective of HLSC treatment was to evaluate short- and long-term clinical, biochemical outcomes, and the maintenance of patient metabolic stability in view of liver transplantation.

## Material and Methods

### Isolation, Culture and Characterization of HLSCs

**Fig. 1:**
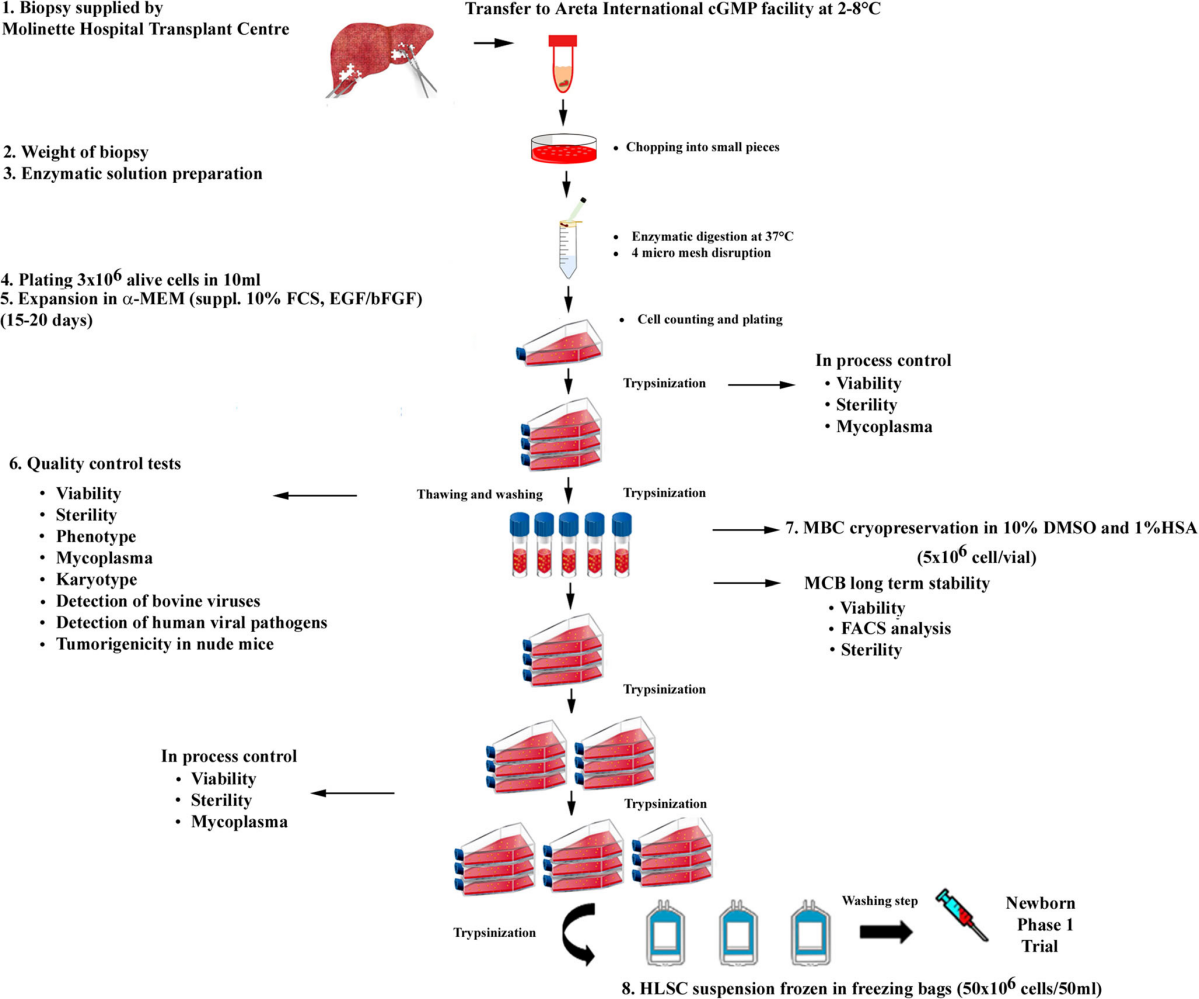
HLSC-master cell bank generation, expansion, collection and storage of cellular suspension protocol in neonatal-onset hyperammonemia Phase I study

The study was approved by the Agenzia Italiana del Farmaco (AIFA) on the basis of approvals the local ethics committee and the Italian Institute of Health as an open-label, prospective, uncontrolled, monocentric Phase I study (HLSC 01–11, EudraCT-No. 2012–002120-33). HLSCs were manufactured according to the requirements of the Directive 2001/20/EC by Areta international (Gerenzano, Italy). The HLSCs master cell bank was obtained from a donor liver belonging to the category of standard risk, as described in the Italian National Transplant Centre Guidelines (batch n° SL-13-001, retest date: November 2015; batch n° SL-13-001, retest date: December 2015; batch n° SL-15-001, retest date: March 2018; batch n° SL-15-002, retest date: March 2018). A complete record of the batch numbers and expiry dates of the study drug was maintained in the Trial Master File. Figure 1 depict the sequential steps involve in the generation of the GMP master cell bank and the final product. The validation of the mater cell bank has been detailed described in the Investigational Medicinal Product Dossier (IMPD) presented to the regulatory authority (AIFA) to obtain the approval of the study. The HLSC master cell bank was generated from a human liver fragment by a modification of the technic previously described for the generation of the research master cell banks [11]. Briefly, the liver biopsy was digested in a solution of GMP-grade collagenase NBI 0.6 mg/ml and 0.73 mg/ml neutral protease NB (both from Nordmark Arzneimittel GMBH & CO.KG, Germany) dissolved in HBSS (Lonza, Basel, Switzerland) in the presence of 3 mM CaCl_2_. After 2 weeks of culture, HLSC colonies were evident and cells were split and expanded in T175 (Greiner S.p.A, Lombardia, Italy). The medium used was alpha-MEM (Lonza) supplemented with 10% gamma irradiated and inactivated GMP-grade fetal calf serum (Lonza), with 2 mM L-glutamine, 4 ng/ml human recombinant GMP-grade EGF (R&D systems, Abington, UK) and with human recombinant GMP-grade FGF-2 (Cellgenix GmbH, Freiburg, Germany).

HLSCs were characterized by indirect immunofluorescence as previously described [11]. Briefly, cells were cultured on chamber slides (Nalge Nunc International, Rochester, NY), fixed in 4% paraformaldehyde and permeabilized with HEPES Triton X-100 buffer. The following primary antibodies were used: anti-albumin, anti-α-fetoprotein (R&D Systems, Abington, U.K), anti-vimentin, (Sigma-Aldrich, St. Louis, MO), anti-nestin (Santa Cruz Biotechnology, CA, USA), anti-nanog, anti-Oct3/4, anti-cytokeratin-8, anti-SSEA4 (all from Abcam, Cambridge, UK), and anti-cytokeratin-19 (Santa Cruz). Alexa Fluor 488 anti-mouse IgG and Texas Red anti-rabbit IgG (Molecular Probes, Leiden, The Netherlands) were used as secondary antibodies. Confocal microscopy analysis was performed using a Zeiss LSM 5 Pascal Model Confocal Microscope (Carl Zeiss International, Jena, Germany). Hoechst 33258 dye (Sigma) was added for nuclear staining.

Cytofluorimetric analyses of HLSCs was performed using the following antibodies phycoerythrin (PE) - or fluorescein isothiocyanate (FITC) -conjugated: anti-CD105, -CD29, -CD73 (all from Miltenyi Biotec) and -CD45, -CD34 (BD Biosciences Pharmingen, San Jose, CA) and albumin (LSBio, LifeSpan BioSciences, Washington, USA). Population percentages and numbers were generated for gated populations from each experiment using Guava easyCyte Flow Cytometer (Millipore, Billerica, MA, USA) and analysed with InCyte software.

### In Vitro Potency Test

To test the potency of HLSC master cell bank to differentiate into hepatocytes, HLSCs were cultured under a condition of microgravity in the Rotary Cell Culture System (RCCS) as described [11]. The production of urea in the supernatants was evaluated after 4 days using QuantiChrom Urea assay kit (BioAssay Systems, CA, USA). In this system, cells were placed in vessels that horizontally rotate about their axis at 8–10 rotations per minute. Cells were cultured in RCCS at a concentration of 250,000 per millilitre in the presence of 60% DMEM low glucose (Thermo Fisher Scientific, Waltham, MA, USA), 40% MCDB-201, 1x insulin-transferrin-selenium, 1x linoleic acid 2-phosphate, 10^−9^ M dexametasone, 10^−4^ ascorbic acid 2-phosphate, hepatocyte growth factor (HGF) (10 ng/ml), fibroblast growth factor 4 (FGF4) (10 ng/ml) all from Sigma-Aldrich and 2% FBS (Invitrogen, Carlsbad, CA, USA). As positive controls human hepatocyte obtained by Lonza were used. As negative control, HLSC-derived from a patient with argininosuccinate synthase deficiency and HLSC cultured in adhesion in T-flask were used. The cell line that was previously characterized expressed the same phenotype and differentiation capabilities of HLSC derived from normal subjects but it has a defective urea production [14].

### In Vitro Osteogenic and Adipogenic Differentiation

HLSCs derived from the master cell bank were culture in Osteogenic Differentiation BulletKit™ Medium (Lonza, Basel, Switzerland) to induce osteogenic differentiation into mature, functionally active osteoblasts as previously described [11]. HLSCs adipogenic differentiation was also evaluated by culturing the cells in Adipogenic Differentiation Medium BulletKit™ Medium (Lonza) which includes both the basal media for induction and maintenance and the necessary supplements for both. Human mesenchymal stem cells (Lonza) were used as positive control.

### HLSCs Preparation and Dose Calculation

HLSCs were supplied in EVA (Ethylene Vinyl Acetate) freezing bags containing 50 × 10^6^ cells/10 ml. HLSCs were thawed at the Cell Factory of the Centro Trapianti Cellule Staminali e Terapie Cellulari of the Ospedale Infantile Regina Margherita and immediately washed in thawing solution (5% albumin in a 0.9% NaCl physiological solution) to reduce DMSO concentration in the suspension to a value below 0.2% (20 mg/ml), within the limits of acceptability established by the applicable regulations (ICH Topic Q3C (R4) “Impurities: Guideline for Residual Solvents”). The whole procedure was performed in class A environment, according to a validated protocol for HLSCs processing and thawing (Protocol PV-PM-HLSC rev.01_04.03.2013 and Report RV-PM-HLSC Rev. rev 01_24-04-2013), and to the standard operating procedure “POS.D.1.03”. Based on preclinical results, 250,000 HLSCs x gram of liver (equal to about 44 × 10^6^ cells for a human newborn bearing a 175 g liver) were assumed to be the No Observed Adverse Effect Level (NOAEL) in rodents [12]. The recommended starting dose was established considering the good safety profile of the treatment and previous experiences with liver transplatation; therefore, a further safety factor of 50% was applied to the NOAEL dose level.

### Study Design and Setting

This was an open-label, prospective, uncontrolled, monocentric Phase I study aimed on assessing the safety of intrahepatic administration of HLSCs in patients with inherited neonatal-onset hyperammonemia (HLSCS 01–11, EudraCT-No. 2012–002120-33). The enrolment period went from January 2014 to December 2016. The study was conducted by the University Hospital City of Health and Science of Turin, Italy (Azienda Ospedaliero-Universitaria Città della Salute e della Scienza di Torino) as non-profit sponsor.

### Study Inclusion and Exclusion Criteria

Patients with neonatal-onset hyperammonemic encephalopathy due to UCDs (i.e. deficiency of carbamylphosphate synthetase I, ornithine transcarbamylase, argininosuccinate synthetase, and argininosuccinate lyase) or OAs (i.e. deficiency of propionyl-CoA carboxylase or methylmalonyl-CoA mutase) were eligible for the study. Additional inclusion criteria were: 1) patients had to be referred to the Department of Pediatrics, being referred to the “Regina Margherita” Children Hospital, University Hospital City of Health and Science, Turin;; 2) a favorable assessment of early liver transplantation; 3) normal liver parenchyma and circulation as evaluated by echography and Doppler ultrasound and 4) parental written informed consent. Exclusion criteria were: 1) uncontrolled coagulopathy;2) concurrent diseases or conditions potentially hampering compliance to the study protocol. For this Phase 1 study, three patients were allowed for enrolment by AIFA.

### HLSCs Administration Protocol

Each enrolled, clinically stable patient was scheduled to receive two HLSC intrahepatic injections (once a week for two consecutive weeks). Standard medical treatment was not discontinued. After standardized thawing and washing (see above), resuspended HLSCs in thawing solution at a concentration of 5 × 10^6^ cells/ml were ready for infusion. Two dosage standards were applied according to the patients’ enrolment order. The first patient (patient 1) received 125,000 cells x gram of liver (about 22 × 10^6^ cells) at each administration. Doubled doses were scheduled in patients 2 and 3, both receiving 250,000 cells x gram of liver (about 44 × 10^6^ cells) at each administration. The HLSCs were percutaneously injected into the liver parenchyma under ultrasound guidance and local anaesthesia. All the procedure were performed in the “Regina Margherita” Children Hospital Intensive Care Unit, University Hospital City of Health and Science. Precautionary hospitalization of two weeks after the first HLSCs administration was scheduled for all patients. During and after HLSCs administrations, the patients were monitored continuously for vital signs (temperature, heart and respiratory rates, blood pressure, and oxygen saturation). Liver parenchyma and circulation were monitored by echography and Doppler ultrasound, respectively. No immunosuppression was scheduled during or after HLSCs administrations, according to the evidence of HLSC immunomodulatory activity [15].

### Primary (Safety), Secondary (Efficacy) Clinical, and Biochemical Endpoints

The evaluation of the safety of intrahepatic HLSC administration in infants with inherited neonatal-onset hyperammonemia was the primary endpoint of this study. After basal assessment (V0), safety data were collected daily and reviewed one week after the first HLSC administration (V1) and one (V2) and two weeks (V3) after the second administration (acute safety). All patients were followed-up until present (long-term safety). All the patients underwent clinical, biochemical, and imaging surveillance addressed to the detection of hepatic and/or extra-hepatic complications, including portal vein thrombosis, intrahepatic hematoma, injury of the hepatic artery, fistulisation (arterial-portal, portal-biliary or arterial-biliary), hepatic injury and failure, liver nodular lesions, pulmonary embolism, abdominal and/or chest haemorrhage, sepsis and any other adverse reaction potentially dependent on the experimental procedures. Histological analysis of native livers explanted at liver transplantation time was performed.

Along with the safety assessments, different clinical and metabolic efficacy variables were also longitudinally evaluated as secondary endpoints. The morbidity rate (defined as the number of metabolic decompensations after HLSC therapy) and the variation in natural protein dietary intake were the main measures of clinical efficacy. Longitudinal assessments of ammonia and disease-specific biochemical markers after HLSC therapy were regarded as metabolic efficacy variables.

## Results

### HLSC Characterization

**Fig. 2:**
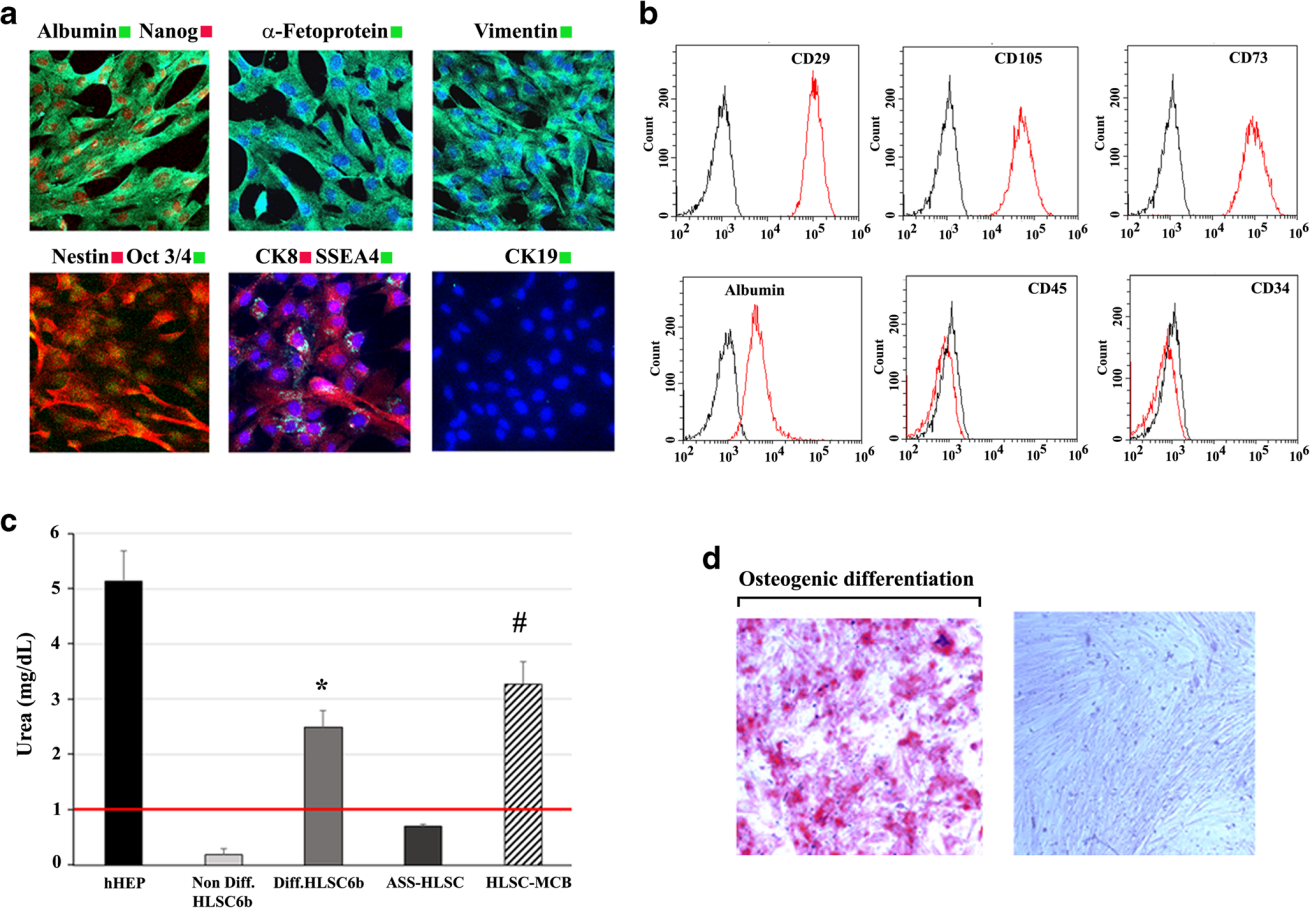
**Characterization of HLSC-master cell bank. (a)** Representative confocal micrographs showing the expression of several hepatic, mesenchymal and embryonic stem cell markers (Original magnification at ×400). **(b)** Representative FACS analyses of HLSCs showing the expression of albumin, CD105, CD29, CD73, albumin, CD45 and CD34 (red histogram). Yellow histograms represent isotypic control. Three experiments were performed with similar results. **(c)** Comparison of urea production by the GMP master cell bank derived HLSCs with HLSC derived from a research cell bank (HLSC6b) and HLSC derived from argininosuccinate synthase deficient liver (HLSC-ASS) in RCCS. Human hepatocyte were used as positive control, undifferentiated HLSC6b (cultured in adhesion) were used as negative control Data are expressed as mg/dL and as mean ± SD of three different experiments. ANOVA was performed; *p < 0.05 HLSCs cultured in RCCS vs HLSCs cultured in adhesion, #p < 0.05 GMP master cell bank derived HLSC vs HLSC-ASS**.** Three experiments were performed with similar results. **(d**) Osteogenic differentiation: left panel, positive staining for calcium deposition as shown by alizarin red staining after 21 days of culture in osteogenic differentiation medium. Right panel, negative staining for alizarin red in HLSC cultured in basal medium (original magnification ×250). Data represent one of three experiments performed with similar results

HLSCs expanded from the master cell bank (passage 7) were characterized according to the criteria previously published [11, 12, 16]. As shown in Fig. 2a, 100% of HLSCs expressed human albumin, α-fetoprotein, and the stem cells markers vimentin and nestin as seen by immunocytochemistry. Moreover, approximately 30% of HLSCs expressed also cytokeratin 8 but all the cells were negative for the oval cell marker cytokeratin 19. As previously described, 100% of HLSCs expressed Nanog, Oct3/4, SSEA4, (Fig. 2a) and Sox2 and Musashi1 (not shown) embryonic stem cells markers. By FACS analysis, HLSCs were positive for mesenchymal markers CD105, CD29 and CD73 but were negative for the hematopoietic markers CD45 and CD34. The expression of albumin was also detected by FACS analysis (Fig. 2b).

As previously demonstrated for the research batches of HLSCs [11], the GMP produced cells were shown to differentiated into mature hepatocytes with production and secretion of urea when cultured in hypo gravity conditions (Fig. 2c). This test was used as a potency assay for the release of the three HLSC batches. Moreover, HLSCs were shown to undergo in appropriated culture conditions into osteogenic differentiation (Fig. 2d) similarly to mesenchymal stem cells but not into adipogenic differentiation at variance of mesenchymal stem cells (not shown). Chromosomal stability, absence of tumorigenicity, viral and endotoxin negativity of the master cell bank are reported in the IMPD. IMPD also reported toxicology, immunomodulatory, dosing, pharmacodynamics and bio distribution studies performed in mice and rats using the intra-liver administration mimicking conditions used for patient treatments.

### Patient Enrolment and Treatment

**Fig. 3:**
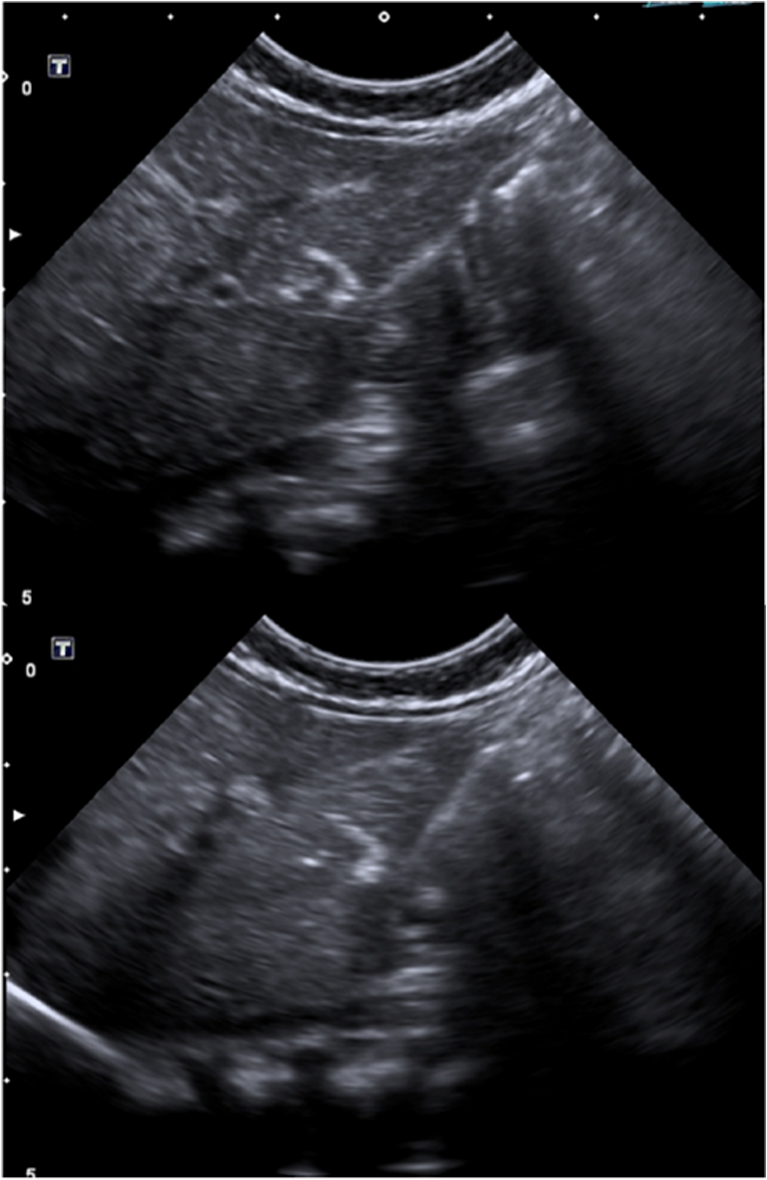
Ultrasound-guided percutaneous intrahepatic injection of human liver stem cells (HLSCs) in an infant with neonatal-onset inherited hyperammonemia while in stable metabolic conditions. HLSCs injection and real-time ultrasound images are presented

**Fig. 4:**
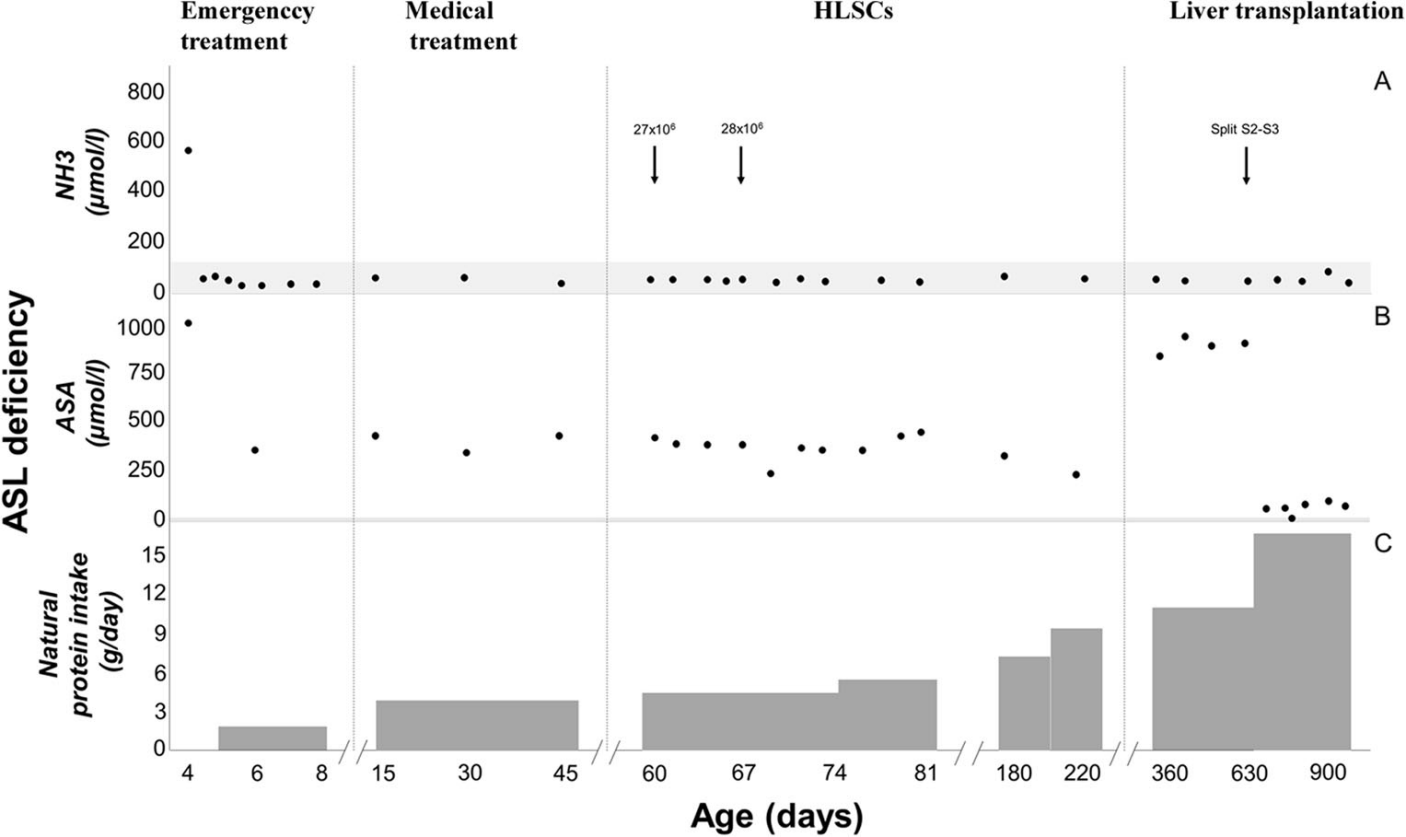
Time course of ammonia (NH_3_), argininosuccinic acid (ASA), and natural protein intake in the first enrolled patient affected by arginine succinate lyase (ASL) deficiency treated with intrahepatic administrations of human liver stem cells (HLSCs)

**Fig. 5:**
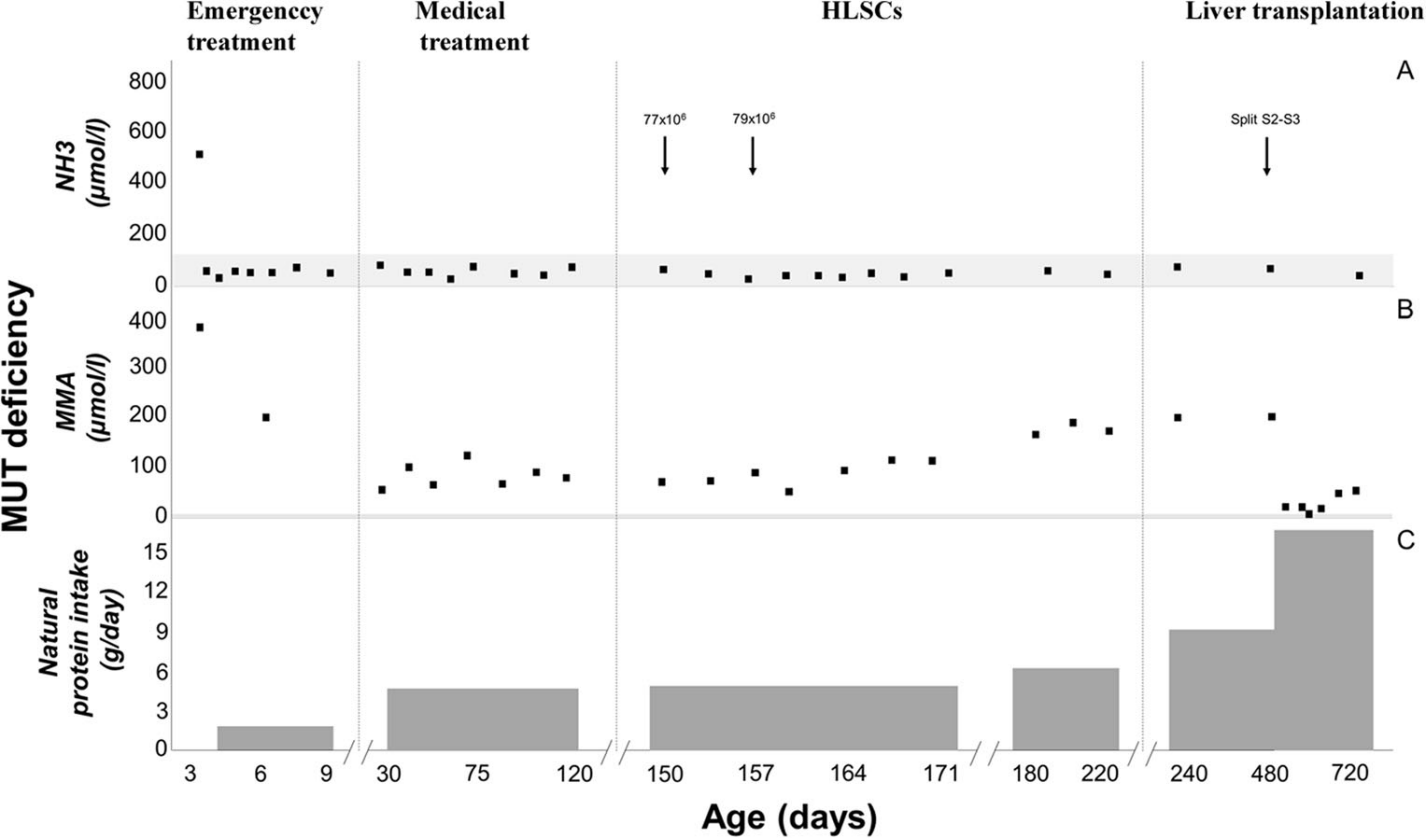
Time course of ammonia (NH_3_), methylmalonic acid (MMA), and natural protein intake in the second enrolled patient affected by methylmalonic acidemia due to methylmalonyl-CoA mutase (MUT) deficiency treated with intrahepatic administrations of human liver stem cells (HLSCs)

**Fig. 6:**
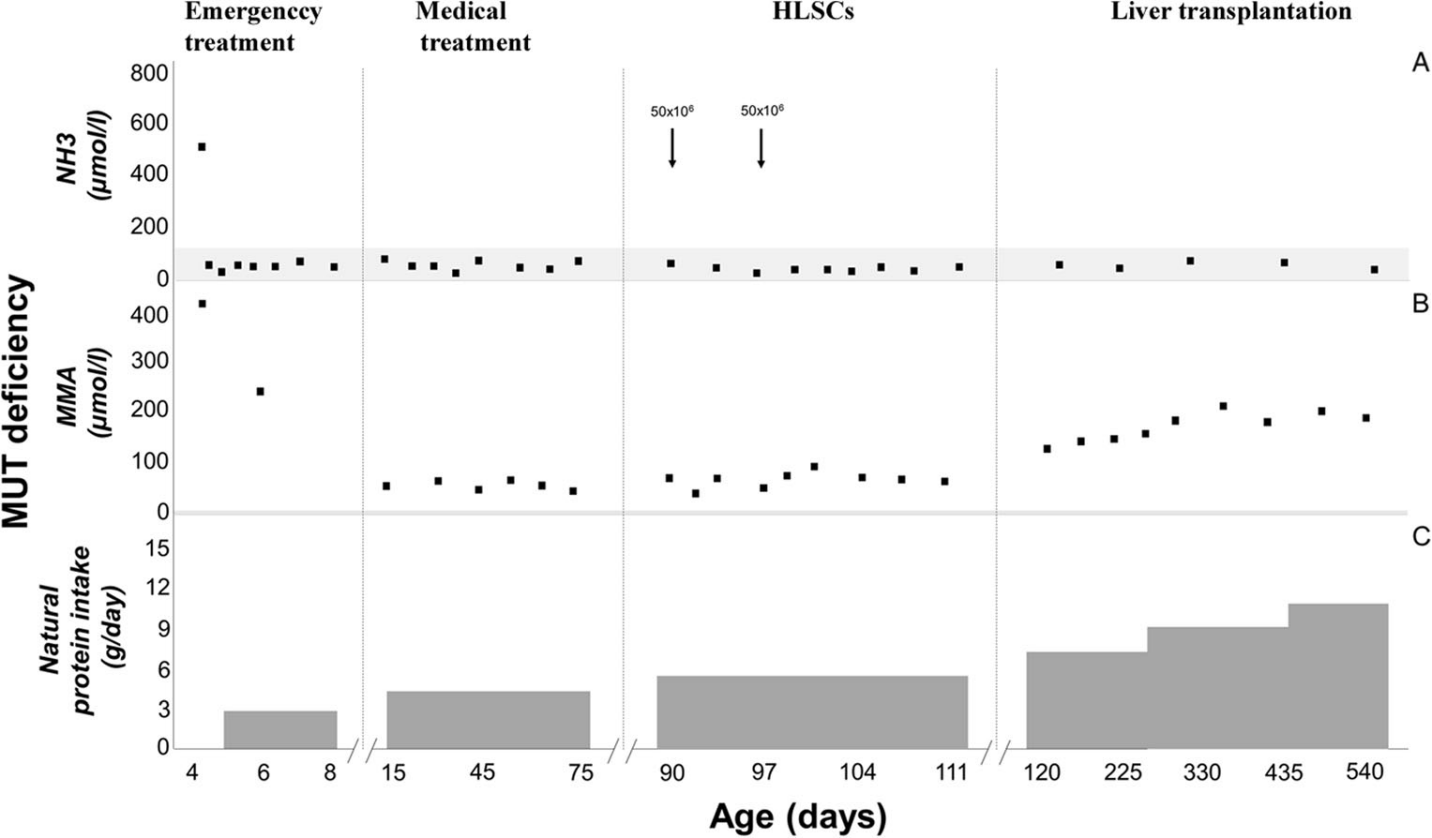
Time course of ammonia (NH_3_), methylmalonic acid (MMA), and natural protein intake in the third enrolled patient affected by methylmalonic acidemia due to methylmalonyl-CoA mutase (MUT) deficiency treated with intrahepatic administrations of human liver stem cells (HLSCs)

During the enrolment period, three patients were diagnosed with inherited neonatal-onset hyperammonemia at the study Center. As they met the inclusion criteria, all these three consecutive patients were enrolled into the study and completed the study protocol. Details on patients’ characteristics and HLSC treatment are provided in Table 1. The volumes required for the administration of the scheduled HLSC dosages by ultrasound-guided percutaneous administrations of HLSCs (Fig. 3) ranged from 5 ml to 11 ml per dose. In all patients, each HLSC injection lasted less than 10 min. No injection-related complications were observed. Details of acute safety outcomes after the HLSC administration in the three patients are provided in Table 2. No deaths, adverse events, intrahepatic or extrahepatic complications and no safety issues were registered at long-term follow-up. After HLSCs administration, no signs of infections and no hyperammonemia metabolic decompensations were recorded in any of the three patients. In particular, steadily normal ammonia concentrations were maintained in spite of a ~30% increase in natural protein daily intake (Figs. 4, 5, and 6, panel A and C). Peripheral concentrations of argininosuccinic acid (ASA) in the patient with argininosuccinic aciduria and methylmalonic acid (MMA) in patients with methylmalonic acidemia were stable after HLSCs injections (Figs. 4, 5, and 6, panel B). After this plateau, a progressive increase of ASA and MMA was invariably observed at the longitudinal follow-up (Figs. 4, 5, and 6, panel B). After liver transplantation in patient 1 and 2, ASA and MMA were markedly reduced (Figs. 4, 5, and 6, panel B). Patients 1 and 2 had liver transplantation after 19 and 11 months from HLSCs administration, respectively, with no surgical complications. After transplantation, both patients had immunosuppressive regimen, which included basiliximab preoperatively and on the fourth day and tacrolimus, using therapeutic drug monitoring to reach the standard target range. The post-transplantation course was complicated in patient 1 by two moderate acute rejections 30 days and 9 months after transplantation, as well as the presence of donor-specific antibodies (DSA) against the transplanted liver (anti DQ7 with mean fluorescence intensity of 16,700), but not against HLSCs. After the second episode of rejection, mycophenolate mofetil was added with complete normalization of liver picture. The post-transplant course in patient 2 was complicated by monomorphic Epstein Barr virus infection correlated with post-transplant lymphoproliferative disorder, successfully treated by rituximab. No DSA antibodies were detected in this patient (against either the transplanted liver or the HLSCs). Extensive histopathological analyses of native livers removed at transplantation revealed normal parenchyma and the absence of nodular lesions and it was not possible to identify the exact site of HLSC injection.

**Table 1:** Characteristics of three patients with inherited neonatal-onset hyperammonemia due to argininosuccinate lyase (ASL) deficiency or methylmalonic acidemia treated with percutaneous intrahepatic administrations of human liver stem cells (HLSCs) as a bridging therapy before liver transplantation (LT)

	Patient 1	Patient 2	Patient 3
Clinical characteristics			
*Diagnosis*	ASL deficiency	Methylmalonic acidemia	Methylmalonic acidemia
*Gene (genotype)*	ASL (c.898G > T/c.913G > A)	MCM (c.655A > T/c.927G > A)	MCM (c.103C > T/c.785G > A)
*Age at onset (days)*	4	3	3
*Peak ammonia (*μmol*/l)*	709	494	757
Neonatal metabolic emergency treatment^*^	yes	yes	yes
*Neonatal renal replacement therapy*	yes	yes	yes
*Age at Liver transplantation (months)*	21	16	waiting list
HLSCs therapy			
*Age at treatment (months)*	2	5	3
*HLSCs dose: 1st administration*	27 × 10^6^	77 × 10^6^	50 × 10^6^
*First sites of injection*	Left hemiliver	Left hemiliver	Left hemiliver
*HLSCs: 2nd administration*	28 × 10^6^	79 × 10^6^	50 × 10^6^
*Second sites of injection*	Right hemiliver	Right hemiliver	Right hemiliver
*Follow-up (months)*	19	11	ongoing

^*^ASL deficiency: protein avoidance, intravenous glucose infusion, sodium benzoate, arginine

^*^MMA: protein avoidance, intravenous glucose infusion, carnitine, vitamin B12

**Table 2:** Basal evaluations (V0) and acute safety outcomes (V1-V3) in one patient affected by argininosuccinate lyase (ASL) deficiency and two patients with methylmalonic acidemia treated by intrahepatic injections of human liver stem cells (HLSCs)

	Patient 1 (ASL deficiency)				Patient 2 and 3 (Methylmalonic acidemia)				All patients			
HLSCs dose	125,000 cells/g of liver				250,000 cells/g of liver							
Visit	V0	V1	V2	V3	V0	V1	V2	V3	V0	V1	V2	V3
Clinical parameters												
*Blood pressure (mmHg)*	91/52	90/52	75/48	72/40	73 ± 23/43 ± 23	90 ± 0/48 ± 4	80 ± 7/52 ± 10	85 ± 7/50 ± 0	79 ± 19/46 ± 17	90 ± 0/49 ± 3	75 ± 9/48 ± 10	80 ± 9/46 ± 5
*Temperature (°C)*	36.1	36.3	36.7	36.0	36.3 ± 0.1	36.3 ± 0.1	36.7 ± 0.7	36.2 ± 0.2	36.1 ± 0.1	36.1 ± 0.6	36.7 ± 0.2	36.1 ± 0.2
*Heart rate*	131	135	116	140	98 ± 2	100 ± 0	97 ± 1	99 ± 0	121 ± 9	137 ± 28	116 ± 24	139 ± 7
*Respiratory rate*	45	55	47	40	55 ± 7	47 ± 18	50 ± 14	54 ± 8	51 ± 7	49 ± 13	47 ± 10	49 ± 10
*Saturation O*_*2*_*(%)*	99	100	98	98	98 ± 2	100 ± 0	97 ± 1	99 ± 0	98 ± 2	100 ± 0	98 ± 2	99 ± 1
Laboratory tests												
*Red blood cells (10*^*12*^*/l)*	4.0	3.9	3.8	4.0	3.6 ± 0.0	4.4 ± 0.4	4.4 ± 0.1	4.4 ± 0.4	3.7 ± 0.2	4.3 ± 0.4	4.2 ± 0.3	4.3 ± 0.3
*Hemoglobin (g/dl)*	10.9	10.0	10.1	10.1	7.9 ± 0.3	10.6 ± 2.4	9.9 ± 0.7	9.5 ± 0.0	8.9 ± 1.7	10.4 ± 1.7	9.9 ± 0.5	9.7 ± 0.3
*Platelets (10*^*9*^*/l)*	720	574	761	458	763 ± 30	484 ± 48	588 ± 186	502 ± 38	749 ± 33	514 ± 62	645 ± 165	487 ± 37
*White blood cells (10*^*9*^*/l)*	12.4	19.8	12.4	11.8	8.8 ± 1.6	7.1 ± 0.3	10.0 ± 3.4	9.1 ± 4.6	10.0 ± 2.2	11.3 ± 7.3	10.8 ± 2.7	10.0 ± 3.6
*INR*	1.1	1.0	1.0	0.9	1.0 ± 0.2	1.0 ± 0.2	1.0 ± 0.2	1.0 ± 0.1	1.1 ± 0.2	1.0 ± 0.1	1.0 ± 0.1	1.0 ± 0.0
*AST (U/l)*	32	37	65	43	41 ± 22	32 ± 4	33 ± 9	37 ± 14	38 ± 16	33 ± 4	43 ± 20	39 ± 10
*ALT (U/l)*	34	33	39	25	39 ± 18	32 ± 4	34 ± 16	37 ± 23	37 ± 13	32 ± 3	36 ± 11	33 ± 18

International normalized ratio (INR, normal range 0.8–1.2); Aspartate aminotransferase (AST, normal range: 20–50 U/l); Alanine aminotransferase (ALT, normal range: 20–50 U/l)

## Discussion

Neonatal-onset UCDs or OAs are life-threatening disorders leading to hyperammonemic coma and requiring early specific emergency treatments [17, 18]. Patients surviving the neonatal period experience recurrent metabolic decompensations later in life (generally triggered by undercurrent catabolic stressors) and poor prognosis in spite of appropriate long-term conventional management [1]. Liver transplantation can improve the clinical outcome of patients, as it is increasingly documented [3–6]. This evidence prompted various clinical studies on the use of hepatocyte transplantation in various inborn errors of metabolism [7–9]. To date, experience with hepatocyte transplantation in 22 patients with UCDs is available [9, 19–25]. In general, hepatocyte transplantation was shown to be transiently effective in restoring the deficient metabolic activity in affected patients, providing the proof of concept of the clinical feasibility of cellular therapies in inborn errors of metabolism. Unfortunately, different issues hamper the clinical use of hepatocyte transplantation. Firstly, hepatocyte transplantation requires invasive procedures for the placement of a catheter into the portal vein, with the risk of dislocation, thrombosis, or other surgical complications [25]. Secondly, hepatocyte transplantation requires relatively high infusion volumes with respect to the available space in the native liver, with an increased risk of extra-liver hepatocyte localization. Thirdly, hepatocyte transplantation invariably requires immunosuppressive regimen to prevent rejection of donor cells and warrant transient metabolic effect [7, 25].

HLSCs were identified as a cell population derived from human adult liver tissue expressing some mesenchymal and some embryonic stem cell markers with multiple differentiation and self-renewing capabilities [11, 12]. The in vitro differentiation into mature hepatocyte was associated with urea production [11]. Moreover, HLSC-derived extracellular vesicles were shown to correct ureagenesis in an in vitro model of argininosuccinate synthetase deficiency [14]. It has not yet been tested whether extracellular vesicles derived from HLSCs may correct ureagenesis deficiency in vivo*.* However, it is possible that few liver engrafted HLSCs may act in a paracrine manner trough extracellular vesicles on nearby enzyme deficient hepatocytes transferring the relevant enzymes. HLSCs were shown to engraft in the liver and to be effective in completely restoring liver function in vivo in a model of fulminant hepatitis developed in non-immunocompetent mice [12]. At variance of oval cells, which have been described in rat and mice but detectable in humans mainly in pathological conditions [26, 27], HLSCs can easily be expanded and cultured from normal adult human liver [16]. Moreover, the evaluation of the telomere length during HLSC expansion from P6 to P17 showed remarkable stability [16]. This evidence prompted us to hypothesize the use of HLSCs as a bridging therapy until liver transplantation in patients with inherited neonatal-onset hyperammonemia.

In this Phase I study, we investigated the clinical safety of HLSC administration in infants with inherited neonatal-onset hyperammonemia due to argininosuccinic aciduria or methylmalonic acidemia. Two main original points characterized the introduction of HLSCs in the clinical setting and facilitated their full safety profile. Firstly, differently from hepatocyte transplantation, HLSCs were administered percutaneously into liver parenchyma under ultrasound guidance and local anaesthesia. This mini-invasive approach was possible because of the small injection-volumes providing the individual HLSCs scheduled doses. Besides avoiding invasive procedures and related complications, the intrahepatic injection procedure theoretically minimizes the risk of pulmonary spreading of transplanted cells compared to portal infusion. Actually, no local or systemic adverse events were registered by this approach. Secondly, no immunosuppressive regimen was planned in patients treated with intrahepatic HLSCs. This was based on the evidence not only of the low immunogenicity of HLSCs, but also on their immunomodulatory abilities that may potentially obviate rejection [15]. Indeed, HLSCs do not express HLA class II or costimulatory molecules CD40, CD80, and CD86. Moreover, HLSCs inhibit T cell proliferation and dendritic cell differentiation as also shown for mesenchymal stem cells [15]. In addition, HLSCs were found to suppress natural killer degranulation protecting them from allogenic lysis [15].

Short- and long-term clinical and biochemical data after HLSCs injections were regarded as secondary endpoints. All patients remained clinically stable and no metabolic decompensations were registered after HLSCs treatment. However, this favourable metabolic control was observed in the absence of undercurrent catabolic stressors (i.e. febrile illnesses), thus making difficult to correlate metabolic stability with HLSC treatment. On the other hand, long-lasting stability of ammonia concentration was maintained in all patients after HLSCs therapy in spite of a consistent increase in dietary protein intake. The ASA metabolic marker of ASL deficiency (patient 1) remained stable up to 220 days whereas the MMA marker of methylmalonic acidemia (patient 2 and 3) remained stable up to 171 and 111 days respectively, after HLSC treatment. However, both these disease specific metabolic markers invariably increased at long-term follow-up reaching maximal levels before liver transplantation. Although the full evaluation of metabolic effectiveness of HLSCs was beyond the objective of this study, the main limitations to this purpose are sample size, disorder heterogeneity and lack of fully reliable functional tools for the assessment of metabolic correction after HLSCs therapy, such as assessment of in vivo ureagenesis not available at the time of the study. Indeed, these issues will be addressed in upcoming studies.

All patients treated with HLSCs were enrolled on the liver transplantation waiting list as from the study protocol and two of them received liver transplanted at 19 (patient 1) and 11 (patient 2) months respectively. The third patient is still in waiting list for transplantation since the stable metabolic conditions. None of the patients developed DSA against HLSCs despite the absence of immunosuppression consistently with the low immunogenicity of the cells. The histological examination of the native livers removed at time of transplantation did not show any parenchymal lesion confirming the bio safety of the cells and of the administration route. The site of injection was not identifiable not allowing identification of HLSCs in the parenchyma. However, the progressive increase of the disease-specific metabolic markers ASA and MMA suggests the absence of long-term persistent HLSC engraftment. Since HLSC can be easily obtainable from a small liver biopsy one can speculate to perform an ex vivo gene therapy to correct the enzyme defect followed by an autologous HLSC transplantation.

In conclusion, we highlighted that percutaneous intrahepatic administration of HLSCs is safe and well tolerated in infants with inherited neonatal-onset hyperammonemia. The full safety profile of this approach was facilitated by avoidance of invasive procedures and immunosuppression. Moreover, this Phase I study indicated that HLSC implantation in the liver did not trigger inflammatory and immune reaction. The potential metabolic effect of HLSCs in inherited and acquired disorders require further studies in a larger cohort of patients and it will be the objective of a next Phase II clinical trial.

## Abbreviations

UCDs: Urea cycle disorders
OAs: Organic acidemias
HLSCs: Human liver stem cells
DSA: Donor specific antibodies
IMPD: Investigational medicinal product dossier
